# Revisiting race 1 of *Pseudomonas syringae* pv. tomato: evolution, effector biology, and host resistance

**DOI:** 10.1128/jb.00494-25

**Published:** 2026-03-18

**Authors:** Miryam Valenzuela, Ariel Herrera-Vásquez

**Affiliations:** 1 Centro de Estudios Avanzados en Fruticultura (CEAF), Millennium Nucleus Bioproducts, Genomics and Environmental Microbiology (BioGEM) 518100 , Rengo, Chile; 2 Centro de Biotecnología Vegetal, Facultad de Ciencias de la Vida, Universidad Andres Bello 682708 , Santiago, Chile; Dartmouth College Geisel School of Medicine, Hanover, New Hampshire, USA

**Keywords:** *Pseudomonas syringae *pv. tomato, race 1, *Solanum lycopersicum*, tomato, bacterial speck, microbial evolution, resistance to pathogens

## Abstract

*Pseudomonas syringae* pv. tomato (Pst), the causal agent of bacterial speck in tomato, is a model for understanding plant-pathogen coevolution. Within this pathosystem, the emergence of race 1 has traditionally been interpreted as a direct adaptive response to the development of Pto/Prf-mediated resistance in tomato. While race 0 strains are recognized through the type III effectors AvrPto and AvrPtoB, race 1 strains evade this immune surveillance by losing, mutating, or silencing these determinants, thereby overcoming Pto-mediated resistance. However, recent genomic and population-level studies reveal that the evolutionary success of a pathogen lineage extends beyond effector loss alone. Diagnostic progress—from differential host assays to genome-informed tools—has refined race discrimination and revealed the clonal dominance of T1-like lineages worldwide. Comparative genomics has uncovered genetic signatures in race 1, including expanded effector repertoires, plasmid-encoded virulence factors, and an abundance of mobile elements that reflect horizontal gene transfer while simultaneously blurring the boundaries of classical race definitions. These features underpin its capacity for immune evasion, host specialization, and global persistence. Recent outbreaks in Chile, North America, and Europe involving highly aggressive T1-like strains suggest an apparent rise in virulence, yet the drivers of this trend remain unresolved. They likely involve a combination of effector diversification, horizontal gene movement, and environmental or agronomic factors. Understanding these processes will require integrative genomic, transcriptomic, and functional approaches to connect genotype with phenotype. Taken together, revisiting Pst race 1 highlights both the utility and the limitations of race-based classifications and underscores the need for genome-informed surveillance and diversified resistance strategies in tomato breeding. More broadly, race 1 provides a valuable model to explore how agricultural selection and genomic plasticity shape pathogen evolution in crop systems.

## INTRODUCTION

*Pseudomonas syringae* is a gram-negative, rod-shaped bacterium that serves as one of the most studied models in plant pathology. Comprising more than 60 pathovars with distinct host ranges, this species complex infects a wide variety of crops and wild plants, causing diseases such as leaf spots, blights, and specks (1, 2). Among these, *P. syringae* pv. tomato (Pst) is the causal agent of bacterial speck of tomato, a disease characterized by dark necrotic lesions surrounded by chlorotic halos on leaves, stems, and fruits. The pathogen thrives under cool and humid conditions, and its dissemination through contaminated seeds, rain splash, and aerosols contributes to recurrent epidemics worldwide (3, 4).

The interaction between *P. syringae* and its hosts exemplifies the molecular arms race that shapes plant-microbe evolution. Pst employs a type III secretion system (T3SS) to deliver a suite of effector proteins into plant cells, manipulating host metabolism and suppressing immunity (2, 5). In response, plants deploy immune receptors that recognize either conserved microbial patterns or specific effector activities, triggering defense responses such as the hypersensitive response (6). This dynamic exchange between virulence and defense has made the tomato-Pst system a cornerstone for understanding the molecular basis of plant immunity.

Within this pathosystem, Pst has diversified into pathotypes—or races—that differ in their ability to infect tomato cultivars carrying specific resistance genes. Two major genotypes are commonly recognized: DC3000-like and T1-like strains, which broadly correspond to the physiological races 0 and 1, respectively. Among these genotypes, race 1 stands out as particularly successful and widespread. It has evolved mechanisms to overcome Pto/Prf-mediated resistance, a key tomato immune system that limits Pst infection, thereby enabling colonization of cultivars that were previously considered immune. The emergence and persistence of race 1 therefore reflect a striking example of adaptive evolution driven by agricultural selection pressure. While historically defined as a discrete race, race 1 now serves as a powerful model to explore how bacterial pathogens evade plant immunity and how genomic plasticity and population-level processes reshape the structure of *P. syringae* in agricultural environments. Although this classification has proven highly useful in both research and breeding contexts, accumulating genomic evidence indicates that the boundaries between these groups are more fluid than originally assumed.

## UNMASKING RACE 1: PHYSIOLOGICAL ASSAYS AND MOLECULAR TOOLS

Accurate identification of Pst race 1 has been essential for understanding pathogen dynamics, tracking epidemiological shifts, and implementing effective resistance breeding programs. Diagnostic strategies typically involve a combination of physiological assays using differential tomato cultivars and molecular techniques targeting effector genes.

**Fig 1:**
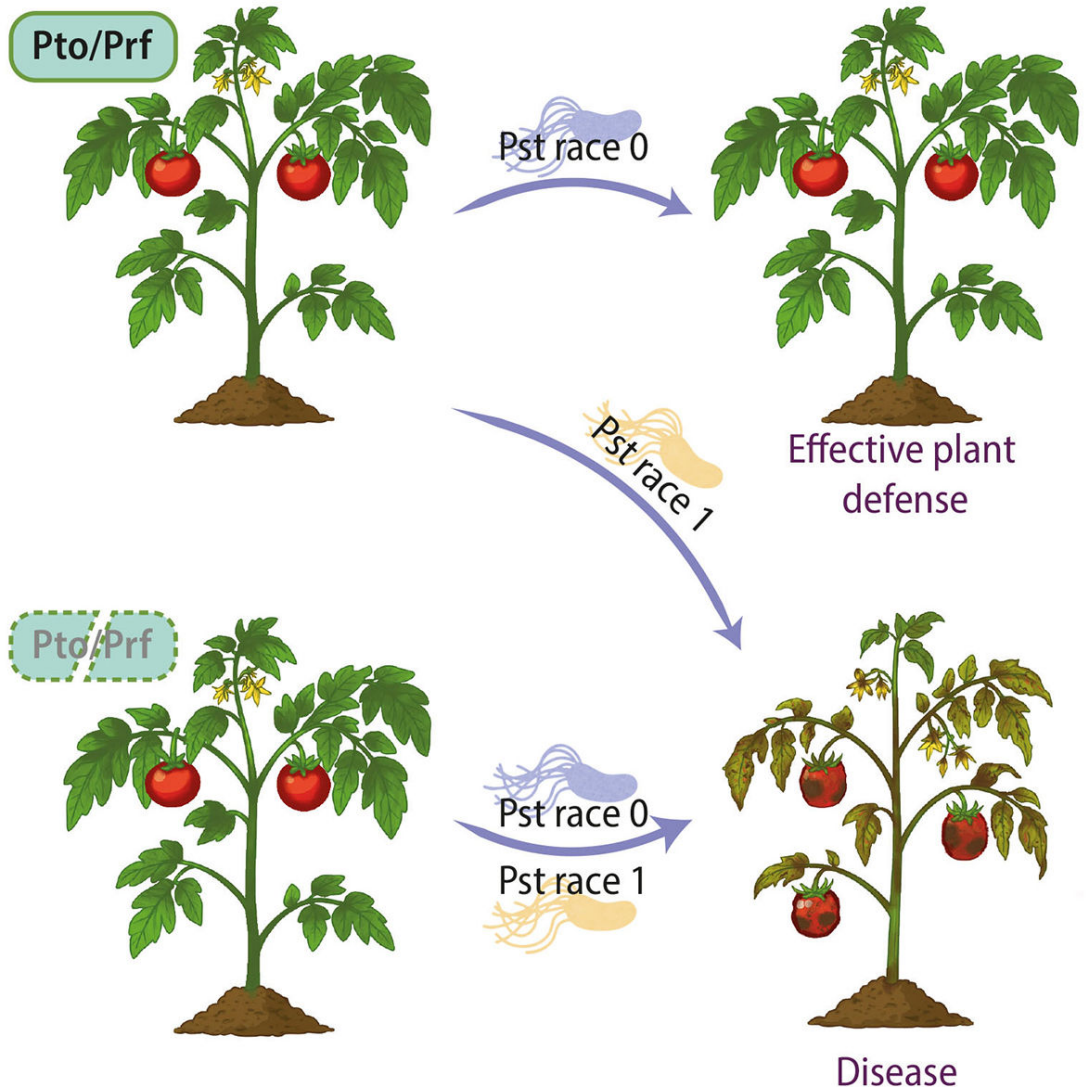
Interaction outcomes between *Pseudomonas syringae* pv. *tomato* races and tomato cultivars differing in the Pto/Prf resistance complex. Tomato plants carrying Pto/Prf (top row) recognize effectors from race 0, triggering an effective plant defense response. Race 1 strains, lacking or inactivating the Pto/Prf-recognized effectors, evade recognition and cause disease. Plants lacking Pto/Prf (bottom row) are susceptible to both races, developing characteristic bacterial speck symptoms.

Physiological diagnosis relies on the differential response of tomato cultivars carrying or lacking the Pto/Prf resistance complex, which is a host immune system that confers resistance to Pst strains expressing the type III effectors (T3Es) AvrPto and/or AvrPtoB (7). Race 0 strains of Pst are avirulent on Pto/Prf-carrying cultivars due to recognition of these effectors by the immune complex, leading to a hypersensitive response that restricts bacterial growth. In contrast, race 1 strains are virulent on susceptible cultivars to race 0 and on Pto/Prf-containing cultivars, as they evade recognition by the immune complex (Fig. 1). Thus, the ability of an isolate to induce disease symptoms on Pto/Prf-bearing tomato cultivars (e.g., “Rio Grande 76R,” “Ontario 7710,” or “Tanya”) has been widely used as a hallmark of race 1. This approach has been employed in several studies from different countries, including the United States (California; 8), Chile (9), and Portugal (10), where race 1 strains have consistently caused disease in the resistant genotypes evaluated.

Complementing pathogenicity tests, molecular diagnostics focus on the detection of effector genes associated with avirulence. PCR-based amplification of the avrPto and avrPtoB genes has been used as a common method to distinguish races. Race 0 strains typically harbor intact versions of both genes, which are normally expressed, while race 1 isolates often exhibit deletions, truncations, or mutations in one or both loci. For example, Kunkeaw et al. (11) used PCR to confirm the absence or non-functional nature of these effectors in field isolates, supporting their classification as race 1. However, the presence of these effector genes is not always sufficient to predict phenotypic behavior, as their contribution depends on proper expression and functional deployment during infection. This limitation has been illustrated by analyses of AvrPto expression levels, assayed by immunoblotting or RT-qPCR, which can distinguish between races even when effector genes are retained, as in the case of New York isolates, which carried both effectors but expressed AvrPto at undetectable levels (12).

**Fig 2:**
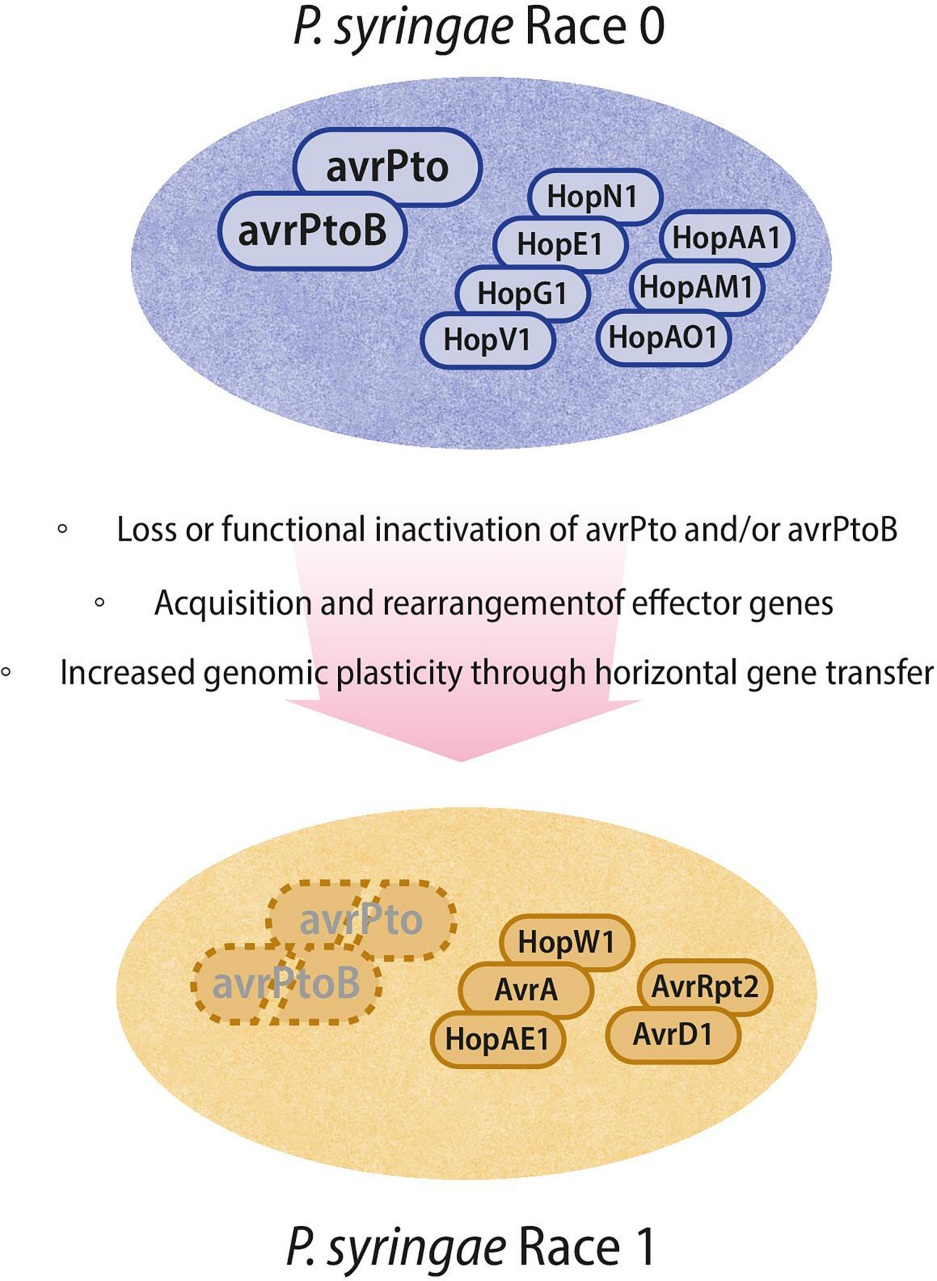
Conceptual model illustrating the evolutionary transition from *Pseudomonas syringae* pv. tomato (Pst) race 0 to race 1 through effector loss, acquisition, and genomic plasticity. Race 0 strains (e.g., DC3000) typically harbor *avrPto* and *avrPtoB*, which are recognized by the tomato Pto/Prf complex, resulting in effector-triggered immunity and restricted infection. Race 1 (T1-like) strains have lost or functionally inactivated these genes and exhibit altered effector repertoires shaped by horizontal gene transfer, genomic rearrangements, and regulatory divergence. The effectors shown (e.g., *avrA*, *hopW1*, *hopAE1*, and *avrRpt2*) represent examples reported in specific strains (14, 15) and are not intended as defining markers of race identity. These changes illustrate broader evolutionary processes underlying immune evasion and host specialization rather than a fixed effector-based race definition.

Building upon these molecular approaches, genome-informed diagnostics have been developed to distinguish highly virulent Pst strains more accurately. Comparative analyses of race 1 and race 0 genomes revealed genetic markers frequently associated with these groups, particularly the presence of *avrA* and *hopW1* in highly virulent T1-like strains and *hopN1* in less virulent DC3000-like isolates (Fig. 2). These findings led to the design of multiplex PCR assays capable of discriminating between high- and low-virulence Pst populations directly from infected tomato tissues. Such genome-assisted diagnostic tools have greatly improved the precision and speed of race identification in field and laboratory settings, supporting more effective epidemiological monitoring and disease management (13).

Advanced molecular tools, including repetitive-sequence-based PCR (rep-PCR), fragment length polymorphism (FLP) analysis, and multilocus sequence typing (MLST), have been used to trace the genotypes associated with race 1. These analyses revealed that most race 1 strains belong to the globally dominant T1 lineage, a clonal group first defined by the strain Pst T1 isolated in Canada in 1986 (16), which lacks functional avrPto/avrPtoB and has effectively replaced race 0 in many production systems (11, 14, 17).

## FROM REGIONAL OUTBREAKS TO GLOBAL DOMINANCE: THE SUCCESS OF RACE 1 IN PST

The rapid expansion of Pst race 1 from localized emergence to global dominance reflects a strong selective advantage under agricultural conditions. This trajectory was first documented in southwestern Ontario, Canada, where field surveys summarized by Lawton and MacNeill (16) detected race 1 at low frequency (about 2% of sampled fields), followed by an increase to 14% within a few years and subsequent detection in nearly 100% of surveyed fields, consistent with strong positive selection in the field (16).

A similar trajectory was subsequently observed in other tomato-growing regions. In California (USA), bacterial speck outbreaks affecting Pst-resistant cultivars were observed as early as 1993 and were formally attributed to race 1 by the late 1990s (8). Continued monitoring revealed that by the late 2000s, race 1 had completely displaced race 0 populations in sampled fields (11). Parallel reports from Europe, including Italy and later in Portugal, documented severe disease caused by race 1 strains in cultivars carrying Pto/Prf-mediated resistance often associated with substantial yield losses (10, 18).

**Fig 3:**
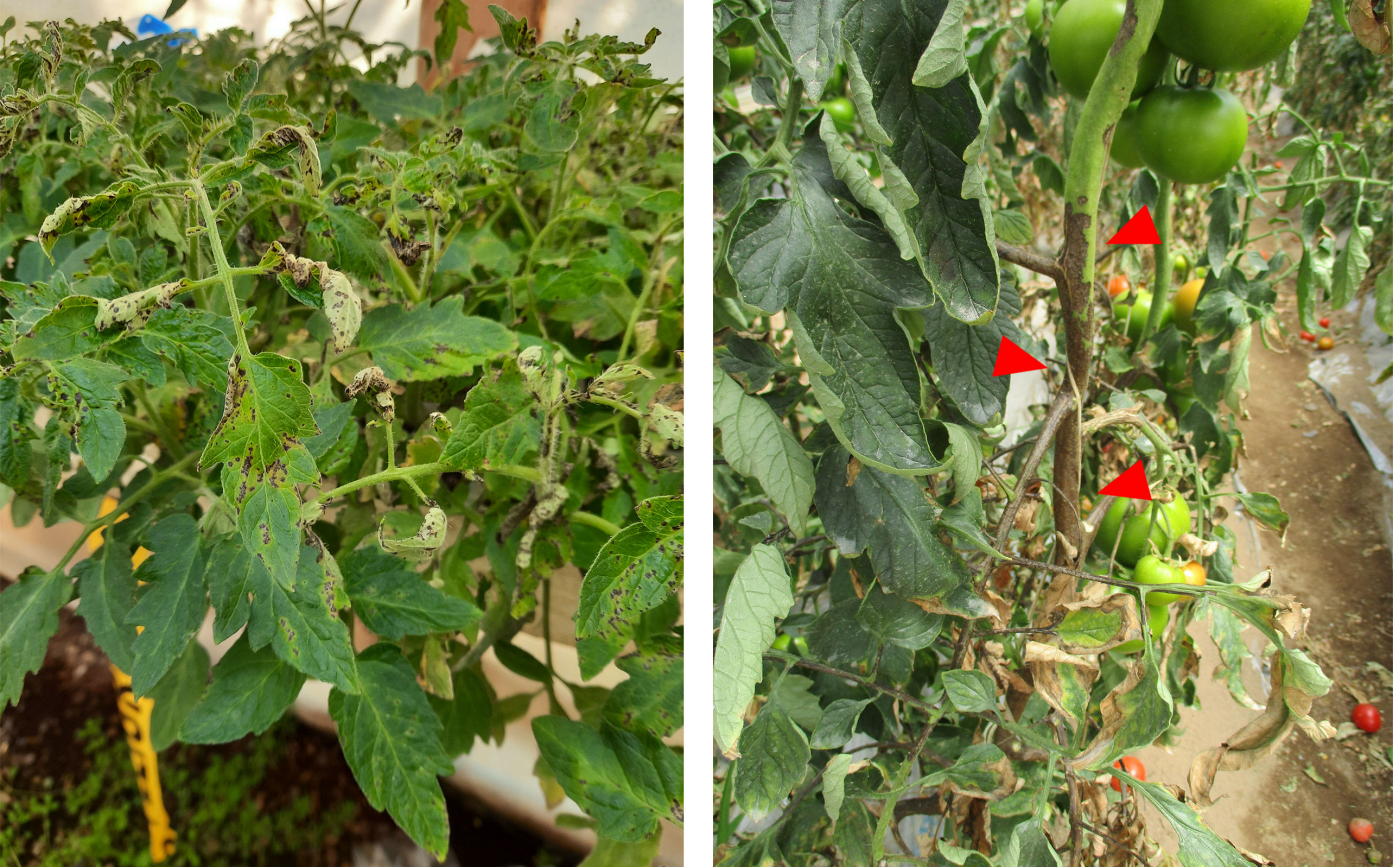
Severe symptoms of bacterial speck on tomato plants (*Solanum lycopersicum*) under field conditions. Leaves exhibit dark brown to black lesions surrounded by yellow halos (left), dark brown to black lesions in the stems, pedicels, and peduncles (right, red arrowheads). The causal agent was identified as *P. syringae* pv. tomato race 1 by housekeeping genes sequencing and pathogenicity assays. The left picture was taken in 2023 in Quillota, Región de Valparaíso, and the right picture was taken in 2015 in Pichidegua, Region de O’Higgins, Chile.

Subsequent outbreaks in diverse geographic regions further underscored the global spread and adaptive success of race 1. In Chile, severe epidemics characterized by stem damage and plant death were attributed to race 1 based on differential host assays and multilocus sequence analyses (9). Importantly, symptoms have continued to be observed in Chilean tomato fields in recent years, even causing the death of young plants (Fig. 3). In Serbia, both race 0 and race 1 strains were detected during the late 2000s, suggesting ongoing population turnover in some regions (19). Notably, isolates from New York displayed intermediate virulence phenotypes, where low AvrPto expression highlighted the plasticity of race-defining traits in natural populations (12).

Early isolations of Pst before the 1960s revealed only DC3000- or JL1065-like genotypes associated with race 0. Over time, strains that possess the ability to overcome *Pto/Prf*-mediated resistance also appeared in countries, including the United States, the United Kingdom, Switzerland, France, Australia, Greece, Venezuela, Italy, Slovakia, Macedonia, Tanzania, and Colombia (17, 20), and T1-like strains began to increase in frequency, demonstrating thereby the widespread presence of Pst genotypes that escape recognition by the Pto/Prf immune complex, displacing previous lineages. Although many of these isolates were not explicitly classified as race 1, they share key molecular traits, such as deletions or functional inactivation of *avrPto* and/or *avrPtoB*, that historically typify this pathotype (20).

Collectively, this historical trajectory highlights not only the geographic expansion of Pst race 1, but also its marked evolutionary advantage over race 0 under field conditions. The widespread deployment of Pto/Prf-mediated resistance has imposed strong directional selection favoring variants capable of evading immune recognition, driving the global dominance of race 1 in many tomato production systems. This success is underpinned by effector loss, acquisition of novel virulence determinants, and extensive genomic plasticity, illustrating the evolutionary consequences of resistance-driven selection in agricultural ecosystems.

## INSIDE THE GENOME: PLASTICITY, HORIZONTAL TRANSFER, AND ADAPTATION

While pathogenicity assays and molecular diagnostics provide practical tools for identifying Pst races, genome sequencing has revealed the genetic innovations that have enabled race 1-associated lineages to thrive. Beyond the well-characterized loss or suppression of *avrPto* and *avrPtoB*, race 1 genomes exhibit a dynamic architecture marked by plasmid-encoded virulence factors and the accumulation of genomic islands and prophages. These features, frequently acquired through horizontal gene transfer, reflect an evolutionary trajectory that promotes immune evasion, host specialization, and ecological success under agricultural conditions. Comparative analyses of race 1 genomes across geographic regions and time points reinforce their overall clonal structure while simultaneously revealing previously underappreciated variability, including hybrid genotypes and mobile avirulence determinants, which complicate race assignments based on phenotypic assays. In this context, genome-level analysis becomes essential—not only to refine race boundaries with greater resolution, but also to uncover the molecular strategies underlying the adaptive success of this pathotype.

Genomic comparisons initially appeared to provide a clear framework to discriminate race 0- and race 1-associated lineages. For example, the comparison between T1, DC3000, and two other *P. syringae* strains revealed a large, conserved core genome shared across different *P. syringae* strains, alongside substantial lineage-specific gene content. Notably, T1 possesses 757 unique proteins compared to the other strains, while about 10% can be associated with mobile genetic elements such as transposons, insertion sequences, or bacteriophages (14). This enrichment in mobile DNA highlights horizontal gene transfer as a major force shaping the accessory genome of race 1-associated lineages and provides a genomic basis for their enhanced adaptability under field conditions. However, the enrichment of mobile genetic elements in T1 highlights that race-associated genomic signatures are shaped by horizontal gene transfer rather than by fixed lineage-defining traits.

Genome-wide polymorphism analyses further illuminate the evolutionary dynamics of race 1-associated lineages. When sequencing reads from geographically diverse T1-like strains—including isolates from Canada, the United Kingdom, France, Italy, and the United States—were aligned to the DC3000 reference genome, more than 11,000 high-confidence single-nucleotide polymorphisms (SNPs) distinguished DC3000 from T1-like genomes. In contrast, fewer than 200 SNPs separated race 1-associated strains from each other, supporting a model of recent clonal expansion following divergence from race 0-associated lineages (17). While this clonal structure supports the epidemiological coherence of race 1-associated populations, it also indicates that phenotypic diversity can emerge within a genetically narrow background, complicating genotype-based race definitions.

Although T1 remains the best-characterized representative of race 1, other genomes have added complexity to our understanding of its diversity. For example, strains A9 and 407 differ in genome size and virulence, with A9 exhibiting higher virulence than 407, despite sharing a conserved set of 27 type III effectors out of 57 described in the *P. syringae* pangenome (21, 22). These observations indicate that virulence variation among closely related strains can arise without major changes in core effector content, highlighting the contribution of accessory genes, regulatory differences, and genomic context to adaptation and field performance. Importantly, this decoupling between effector inventories and pathogenic outcomes challenges the assumption that race or virulence can be inferred directly from gene presence or absence and instead underscores the need to consider how virulence determinants are regulated, mobilized, and deployed in natural populations.

An interesting case highlighting the instability of race boundaries is the strain DAPP-PG 215, initially described as race 0 based on its phenotypic response to Pto/Prf-carrying tomato plants (15). Although its genomic architecture is closely related to T1 and T1-like strains, such as Max13, K40, and NYS-T1, all known race 1 strains, and harbors several race 1-associated markers such as avrA and hopW1, DAPP-PG 215 retains a functional avrPto1 ortholog that confers a race 0 phenotype.

Genome analysis revealed extensive enrichment in mobile genetic elements, including multiple prophages and genomic islands distributed across the chromosome and plasmids, with *avrPto1* itself embedded within a prophage region. These findings demonstrate that key avirulence determinants can be mobilized through horizontal gene transfer, rendering genotype-based race assignments inherently unstable and underscoring the importance of phenotypic context in race classification.

Taken together, these genomic observations do not simply expand our understanding of race 1 diversity but fundamentally challenge how race is interpreted as a biological category. Rather than representing a stable genomic entity, race 1 emerges as a dynamic outcome of mobile genetic elements, regulatory variation, and population-level processes that decouple virulence from fixed effector inventories. This genomic plasticity highlights the limitations of phenotype-only classifications and motivates integrative frameworks that combine pathogenicity assays with genome-scale data. These insights set the stage for reconsidering effector repertoires not as isolated determinants of virulence, but as components of flexible and cooperative pathogenic strategies, which are explored in the following section.

## BEYOND EFFECTOR REPERTOIRES: PLASTICITY, REGULATION, AND COOPERATIVE VIRULENCE IN PST

The evolutionary success of Pst race 1 cannot be fully explained by static differences in T3E repertoires alone. While T3Es are central determinants of host manipulation and immune suppression, accumulating evidence indicates that virulence in Pst emerges from a dynamic interplay between effector composition, regulatory plasticity, horizontal gene transfer, and population-level interactions (14, 15, 17, 23, 24). In this context, race 1 represents not merely a loss-of-recognition phenotype but a broader adaptive strategy framed by genomic plasticity, microbial companion communities, as well as agricultural selection shaping pathogen success.

Comparative phylogenetic and genomic analyses initially provided insight into how closely related Pst lineages diverged in host range and pathogenic behavior. Multilocus sequence typing revealed that DC3000-like strains belong to a phylogenetic group capable of infecting both Solanaceae and Brassicaceae hosts, whereas T1-like strains—historically associated with race 1—are largely restricted to tomato and fail to infect *Arabidopsis thaliana* (20, 25). Despite their close evolutionary relationship, these lineages differ in key virulence-associated traits, illustrating how modest genomic divergence can result in marked differences in host compatibility and disease outcome (14).

At the molecular level, divergence between race 0- and race 1-associated lineages is closely linked to differences in effector deployment rather than to wholesale remodeling of the virulence machinery. Comparative genomic analyses revealed that DC3000 and T1 share a conserved core genome but differ in a limited subset of T3Es that directly influence host recognition and specificity (14). Alterations affecting the presence, structure, or expression of effectors recognized by the Pto/Prf immune complex—most notably AvrPto and AvrPtoB—are sufficient to shift the outcome of infection from recognition to evasion (11, 22). This principle is exemplified by race 1 field populations that retain overall genomic similarity to reference strains yet display enhanced virulence on Pto-containing cultivars through effector loss, truncation, or transcriptional silencing (11).

Genome sequencing has further revealed that immune evasion in race 1 is embedded within a highly dynamic accessory genome shaped by horizontal gene transfer. Race 1-associated lineages are enriched in mobile genetic elements, including genomic islands, prophages, and plasmids, which frequently harbor effector genes and other virulence determinants (14, 15). The localization of key effectors such as *avrA* and *hopW1* within mobile regions provides a mechanistic explanation for the emergence of strains with mixed genomic and phenotypic signatures and underscores the inherent instability of genotype-based race assignments (15).

Effector function is further modulated at the level of gene regulation. Transcriptomic and proteomic analyses have shown that not all encoded T3Es are expressed simultaneously or at comparable levels, and that their deployment can be strongly influenced by environmental conditions, host-derived signals, and bacterial regulatory networks (22). Consequently, virulence phenotypes may arise from differences in effector expression dynamics rather than from gene content alone, adding an additional layer of flexibility to race 1-associated pathogenic strategies.

Recent studies have further challenged the assumption that virulence is an intrinsic property of individual Pst isolates. Instead, accumulating evidence supports a model of cooperative virulence in which T3Es function as shared resources within bacterial populations (23, 24). In this framework, the immunosuppressive effects resulting from effector delivery into host cells can be exploited by neighboring bacteria lacking a complete virulence repertoire, allowing mixed populations of virulent and less virulent strains to collectively cause disease (23). In addition, spatial and temporal coordination of type III secretion and motility among *P. syringae* subpopulations further supports the idea that pathogenic success can emerge from collective behaviors rather than uniform effector deployment across all cells (24).

Taken together, these observations indicate that the adaptive trajectory of Pst race 1 reflects the convergence of effector diversification, regulatory plasticity, horizontal gene transfer, and cooperative population dynamics. While classical race classifications remain valuable for describing specific host-pathogen interactions, the mosaic nature of effector repertoires, the existence of intermediate genotypes, and the contribution of population-level processes reveal important limitations of binary race models (14, 15). Understanding virulence and adaptation in Pst therefore requires integrative frameworks that move beyond effector inventories to incorporate genome context, regulation, and microbial social interactions as key drivers of pathogen evolution under agricultural selection.

## WHAT WE KNOW, WHAT WE DO NOT, AND WHERE TO GO NEXT

Since Pst race 1 was first reported, its detections have increased both locally and globally, displacing the original race 0. Although this shift was initially attributed to the loss or inactivation of avrPto, accumulated genomic and functional evidence indicates that the differences between the described races reflect a broader reconfiguration of their effector and accessory gene repertoires that define distinct evolutionary trajectories (11, 14, 17). It is striking that Pto-mediated resistance to race 0 is found mainly in industrial processing tomato cultivars, while few fresh-market varieties carry this resistance (12). Nevertheless, this limited deployment appears to have exerted sufficient selective pressure to favor the emergence of strains capable of evading Pto/Prf-mediated recognition. This raises an open question: Was Pto-based selection the primary force driving the rise of race 1, or did additional ecological or evolutionary factors—such as bacteriophage dynamics, plasmid mobilization, or changing environmental conditions—also contribute to its diversification and spread?

Another intriguing question concerns the subsequent adaptation of race 1 to its host. The T1 lineage is restricted to tomato and fails to infect *A. thaliana* or other species, suggesting a process of niche specialization (11, 25). This specialization may have facilitated the fine-tuning of virulence traits through genomic plasticity, including the acquisition and rearrangement of effectors, toxins, and regulatory genes. Comparative genomic studies indicate that these adaptive innovations often reside within mobile elements such as prophages, plasmids, and genomic islands (14, 15, 17), supporting the idea that horizontal gene transfer underlies the evolutionary success and ecological persistence of race 1.

Recent field observations further suggest that race 1-associated populations may continue to evolve increased aggressiveness. In Chile, bacterial speck has historically been a secondary concern compared with bacterial canker caused by *Clavibacter michiganensis* (26). However, recent outbreaks causing severe stem necrosis and seedling death indicate that more virulent Pst populations are now established in tomato fields. Similar trends have been reported in North America and Europe, where highly aggressive T1-like strains have emerged (12, 13). While the drivers of this apparent increase in virulence remain unclear, it likely reflects the combined effects of effector diversification, regulatory rewiring, mobile genetic elements, and environmental factors such as temperature shifts, humidity, or agronomic practices.

Addressing these questions will require integrative approaches that move beyond race classifications. Comparative genomics remains essential to identify virulence associated with gene repertoires and their genomic contexts. This genomic information must be integrated with transcriptomic and functional analyses to elucidate the molecular basis of the aggressive symptoms displayed by highly virulent strains and their host specificity. In parallel, emerging evidence for cooperative virulence among *P. syringae* populations highlights the need to consider population-level interactions, plasmid exchange, and phage-mediated gene flow as contributors to pathogenic outcomes in the field.

Finally, the evolutionary trajectory of Pst race 1 underscores the limitations of resistance strategies based on single dominant genes. Encouragingly, wild tomato relatives harbor resistance loci that operate independently of Pto, including quantitative and multigenic mechanisms (27). Future breeding efforts will benefit from integrating diverse resistance sources, genomic-assisted selection, and continuous pathogen surveillance to anticipate evolutionary shifts in Pst populations. In this context, durable disease control emerges not solely as a genetic challenge, but as an ecological and evolutionary one.

In summary, Pst race 1 represents more than a single pathogenic variant—it is a model for understanding how microbial evolution, through genetic innovation, and population-level processes intersect to drive pathogen adaptation under agricultural selection. Elucidating these dynamics across molecular, evolutionary, and ecological scales will be essential not only for managing bacterial speck of tomato, but also for anticipating similar evolutionary trajectories in other crop-pathogen systems.
